# Event and Comment

**Published:** 1914-04-15

**Authors:** 


### DENTAL REGISTER.

Vol. LXVIII. April 15, 1914. No. 4

### EVENT AND COMMENT.

SCHEME TO FINANCE ORAL HYGIENE MOVEMENT. In this issue we print an article by Dr. W. G. Ebersole on a plan to secure money for establishing dental clinics for oral hygiene. The plan is very well conceived and ought to be workable. We are curious to know how the drug trade and the manufacturers of dental toilet articles will take it. It may curtail the sale of such articles for a time in communities where the campaign is run, but it ought to create a larger demand ultimately for all such preparations and, manufacturers and druggists may be willing to help on the cause that ultimately they will receive the greater benefits. Its a big undertaking for some one, but we hope it may succeed.

PREVENTION OF MALOCCLUSION. We are printing in this issue a report of a Clinical Lecture given by Dr. M. T. Watson, on the early treatment of Malocclusion, which it strikes us is so sane and fundamental that every dentist who is interested in preventive dentistry should not only read most carefully, but should keep his eyes open when inspecting the mouths of little patients. In fact it would be a most excellent thing if some cards were printed containing the statement which Dr. Watson makes in regard to the Clinical evidences of future Malocclusion, and these were sent to every practicing dentist that he might be so impressed that he would be able to advise every mother to present her little ones for frequent inspection. In this way a lot of future troubles could be obviated, not only as to Malocclusion with its difficult problems, but the associated nose and throat complications could be obviated. We are not sure but that the whole subject of good health might not be secured by a proper attention to the proper development of the jaws and facial bones. If a child could have perfect teeth with which to masticate its food and a perfect nasal tract for the free admission and proper preparation of all the air taken into the lungs, why should it not have perfect assimilation and consequent growth to the normal in size and capacity. This is certainly a most timely comment and we hope our oral hygienests will take notice of it.

DENTISTRY FOR CHILDREN. We have often asked why it is that most dentists do not care to treat their child patients, and either ignore them entirely or turn them over to the office assistant, unless they are in serious pain and so demand attention. It would seem that, "just as the way the twig is bent so the tree will grow," is as good philosophy in caring for human as for vegetable growths. But we don't act as sensibly about children as we do about pigs and plants. We recently visited a childrens hospital and saw a room full of babies sleeping as quietly and peacefully as a litter of puppies. We asked the physician in charge why some of them were not howling as is usual in the homes. He said one of these babies cried almost continually when at home and did not grow of any consequence during the first two months of its life. After a few days in the hospital it became one of their best behaved babies and he expected it to become a normal baby and to grow as it should; all because it was being properly fed and cared for. The nurses were not fussing the babies all the time and visitors were not allowed to do so either. No medicines were used ordinarily but the babies were placed in healthful and normal conditions. We were duly impressed and we could not help asking why some one did not start a school to teach mothers how to raise healthy babies. What a grand thing it would be if our young women would spend some of the time they now give to learning the "tango" to learning what for them is the most important thing in all the world for them to know. Preventive medicine is the present ambition of our doctors, would not a grander aim for our young women be the development of a race that would have no use for any kind of medicine We have for years had our mind set on the establishment of a childrens hospital and school in which the oral and nasal conditions should be treated both from the rescue and repair standpoints and with the prevention ideals as the standards, believing that if the child could have healthy and normal breathing and chewing functions that he would surely develop into the normal man. If we could have normal motherhood and scientific nursing of the baby we should have little need for a childrens hospital, and as a consequence little need for the "grown ups" hospital, except for the aged and such as have dissipated their good inheritance. Oh, my, but these are great ideals and it may be they are visionary and impracticable. But are they not rational, and may they not become as possible as the telephone, electric light, X-ray, and hundreds of other man discovered mechanical and physical inventions?

Could anything now thought worthy of striving for be compared with such a goal? If Uncle Sam, or Mr. Carnegie would stop spending money for battle ships and peace memorials, and our suffragetts would take the money and start a campaign of Education for Motherhood, the trick could be done.

